# Extracellular vesicles are rapidly purified from human plasma by PRotein Organic Solvent PRecipitation (PROSPR)

**DOI:** 10.1038/srep14664

**Published:** 2015-09-30

**Authors:** Xavier Gallart-Palau, Aida Serra, Andrew See Weng Wong, Sara Sandin, Mitchell K. P. Lai, Christopher P. Chen, Oi Lian Kon, Siu Kwan Sze

**Affiliations:** 1 School of Biological Sciences, Nanyang Technological University, Singapore, 637551; 2 Department of Pharmacology, Yong Loo Lin School of Medicine, National University of Singapore, Singapore; 3 Memory, Aging and Cognition Centre, National University Health System, Singapore; 4 Division of Medical Sciences, Humphrey Oei Institute of Cancer Research, National Cancer Centre Singapore, 11 Hospital Drive, Singapore 169610

## Abstract

Extracellular vesicles (EVs) such as exosomes and microvesicles mediate intercellular communication and regulate a diverse range of crucial biological processes. Host cells that are damaged, infected or transformed release biomarker-containing EVs into the peripheral circulation, where they can be readily accessed for use in diagnostic or prognostic testing. However, current methods of EV isolation from blood plasma are complex and often require relatively large sample volumes, hence are inefficient for widespread use in clinical settings. Here, we report a novel and inexpensive method of rapidly isolating EVs from small volumes of human blood plasma by PRotein Organic Solvent PRecipitation (PROSPR). PROSPR encompasses a rapid three-step protocol to remove soluble proteins from plasma via precipitation in cold acetone, leaving the lipid-encapsulated EVs behind in suspension. This generates higher purity EVs that can then be obtained from filtration or classical ultracentrifugation methods. We foresee that PROSPR-based purification of EVs will significantly accelerate the discovery of new disease biomarkers and the characterization of EVs with potential for clinical applications.

Extracellular vesicles (EVs) are critical mediators of intercellular communication that regulate a diverse range of biological and pathological processes including leukocyte responses, cancer development and neurodegeneration. Host cells that are damaged, infected or transformed release biomarker-containing EVs into the peripheral circulation, where they can readily be accessed for study and potential use in diagnostic or prognostic testing. The characterization of plasma EVs are likely to be paramount to our current understanding of fundamental biological processes and disease pathophysiology. However, current methods of EV isolation have proven ineffective and time-consuming for extensive use in clinical settings^1^. In spite of some progress made in recent years, basic classification of EVs categorized by morphological criteria or protein composition remains highly inaccurate^2^,^3^. Current nomenclature categorizes major EV subtypes as exosomes (30–100 nm vesicles secreted from the cytoplasmic multivesicular body machinery) or as microvesicles (100–1000 nm vesicles secreted from the endosome-like domains of the plasma membrane), which exhibit a heterogeneous composition^4^.

Efficient methods of EV purification from complex biological fluids and tissues such as blood plasma will be vital to enable their characterization and future use in medical practice. Patient samples frequently contain large quantities of soluble proteins, aggregates and contaminants that restrict EV analysis using current ‘omics’ platforms. The most common method of EVs isolation is ultracentrifugation coupled with sucrose gradient or cushion separation^5^,^6^. However, this laborious and time-consuming approach requires large volumes of blood plasma (>2 ml), and often the process results in EV co-separation with unwanted contaminants^1^,^7^,^8^,^9^. More efficient methods of EVs purification are therefore required to accelerate the study of EVs biology and assist the translation of these data into clinical applications.

Here, we hypothesize a more effective approach comprising the removal of soluble proteins from biological samples by precipitation, leaving a supernatant enriched in a heterogeneous population of EVs for further analysis. In our study, we report that the isolation of EVs from small volumes of human blood plasma can be achieved by PRotein Organic Solvent PRecipitation (PROSPR); a fast and simple procedure that could significantly propel technological advances in the study of EVs biological implications and biomarker discovery.

## Results

### Optimization of the PROSPR method for isolation of extracellular vesicles (EVs)

Extracellular vesicles (EVs) are rich in cargoes that may be identified as potential biomarkers of disease processes. However, current methods of isolating EVs from complex biological fluids are inefficient and slow. We thus sought to optimize an alternative approach based on PRotein Organic Solvent Precipitation (PROSPR). We first used healthy control blood plasma to test the ability of three different organic solvents (acetone, chloroform and trichloroacetic acid) to precipitate plasma proteins under similar optimal conditions (1:4 v/v solvent to plasma ratio^10^,^11^,^12^). The purified EVs were further processed for proteomic analysis. The LC-MS/MS analysis of the tryptic-digested proteins contained in the PROSPR-purified EVs revealed that acetone was the most efficient of the three solvents tested (Supplementary Data Set 1). The proteomic profile obtained by chloroform-based PROSPR exhibited a short list of identified proteins and none of the exosomal markers was identified by this method (Supplementary Data Set 2). It is thus likely that EVs may have been deteriorated in chloroform. We also concluded that trichloroacetic acid-based PROSPR was highly complex and inefficient during the evaporation step, and would not contribute to our pursuit for a rapid and efficient isolation method.

**Figure 1:**
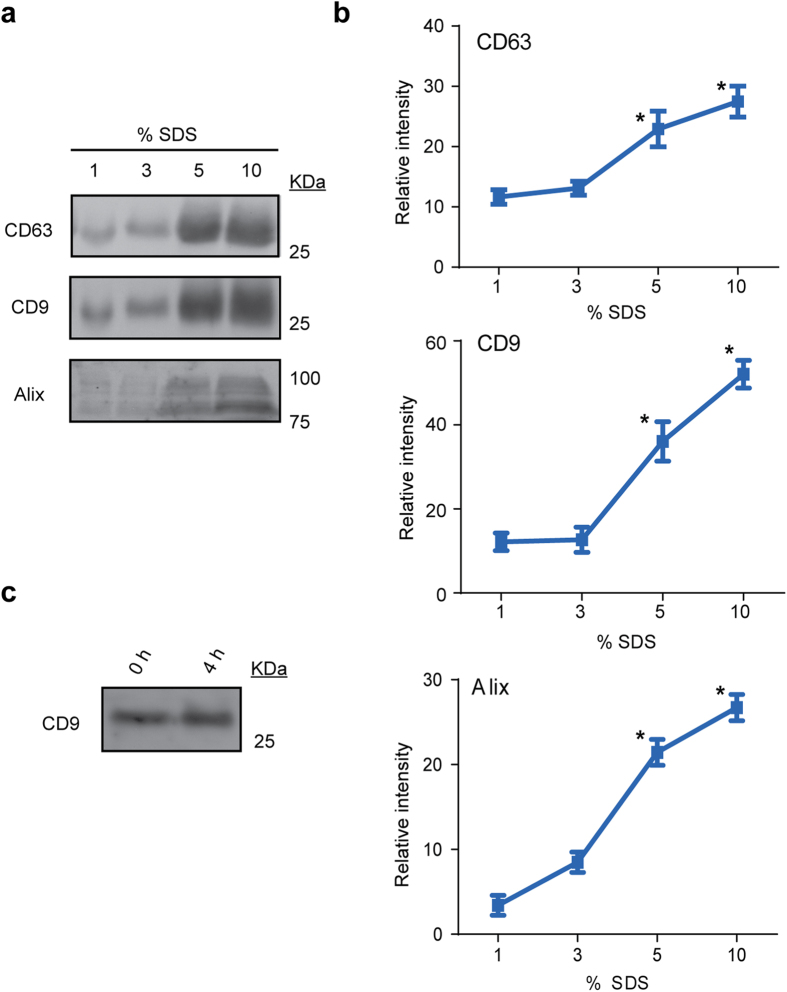
Western blot detection (**a**) and relative quantification (**b**) of characteristic EV markers in PROSPR-separated EV samples after treatment with a range of varying doses of SDS detergent. Significantly higher amounts of CD63, CD9 and Alix proteins were detected at SDS concentrations ≥5% in the lysis buffer (*p < 0.05). The SDS dose-dependent western blots of plasma EVs were performed in triplicate. The stability of EVs was also assessed by western blot (**c**) and no clear differences between the time intervals studied were revealed. These data suggest that the isolated EVs remain stable for at least four hours in the PROSPR buffer and require higher concentrations of solubilizing agent to be properly lysed.

**Figure 2:**
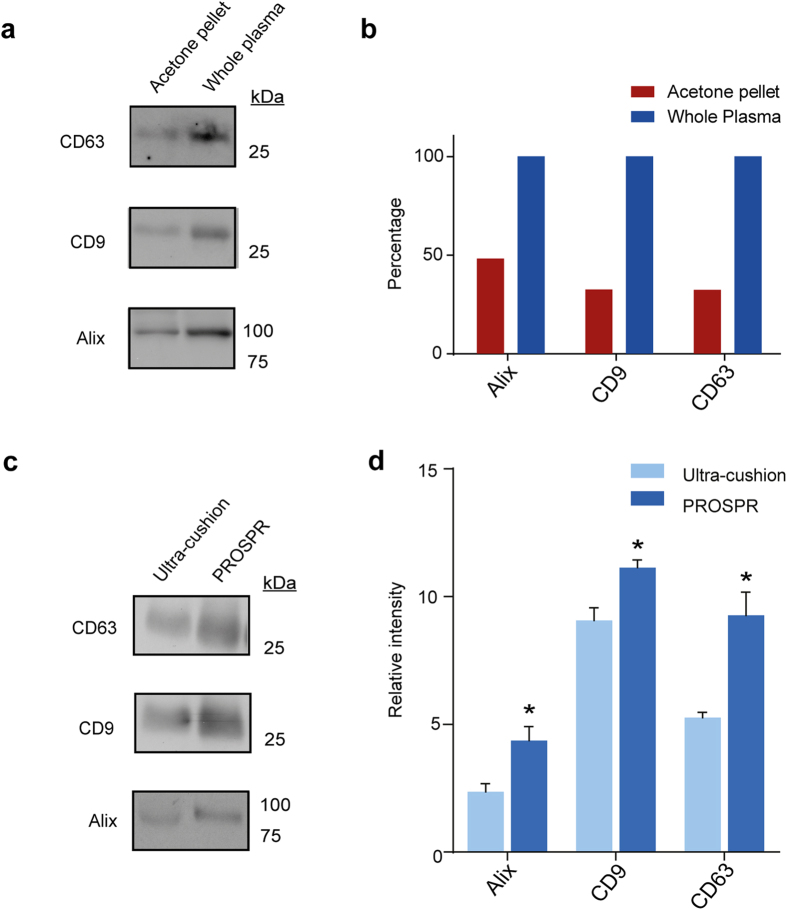
Western blot detection (**a**) and percentage of relative quantification (**b**) of whole plasma and acetone pellet fractions before and after perform PROSPR isolation of EVs. The obtained results show that PROSPR is able to successfully isolate between the 50 and 70 percent of the characteristic EV markers (CD63, CD9 and Alix) present in whole plasma samples. Presence of EV markers was also analyzed in ultra-cushion and PROSPR fractions and the ESCRT adaptor protein Alix and the tetraspanins CD9 and CD63 were used as indicators of purified EVs (**c**). Higher isolation capacity of the PROSPR method (**d**) was achieved for each of the three tested EV markers (*p < 0.05).

Since detailed biochemical analysis of EV contents requires membrane disaggregation and release of vesicular cargoes, we next assessed the efficiency of EV disruption by Western Blot with a range of different SDS concentrations (1–10% SDS in PBS). EV lysis was determined by detection of common EV markers (Fig. 1a,b). We observed that maximal detection of the EV-associated proteins CD9, CD63, and Alix was achieved using SDS concentrations ≥5%. Our data further indicated that higher SDS concentrations were associated with improved release of total EV proteins, consistent with earlier reports that ultracentrifugation-separated EVs include distinct subsets with detergent-resistant properties^13^. We next assessed the stability of the plasma EVs over time when plasma was incubated in PROSPR precipitation buffer at −20 °C up to four hours. Western blot determination of CD9 levels clearly demonstrated that EVs prevailed during the time interval assessed in acetone precipitation buffer, with no significant differences in antigen detection (Fig. 1c). Finally, we assessed the relative yielding profile of PROSPR by western blot (Fig. 2a,b). The results showed that PROSPR isolated between the 50%–70% of the whole amount of EV characteristic antigens from blood plasma. These data collectively suggest that blood plasma EVs can be successfully isolated by acetone-based PROSPR separation and remain stable in PROSPR buffer during at least 4 hours, furthermore these EVs can only be efficiently lysed using high concentrations of denaturing agents.

### Ultrastructural characterization of PROSPR-separated EVs

**Figure 3:**
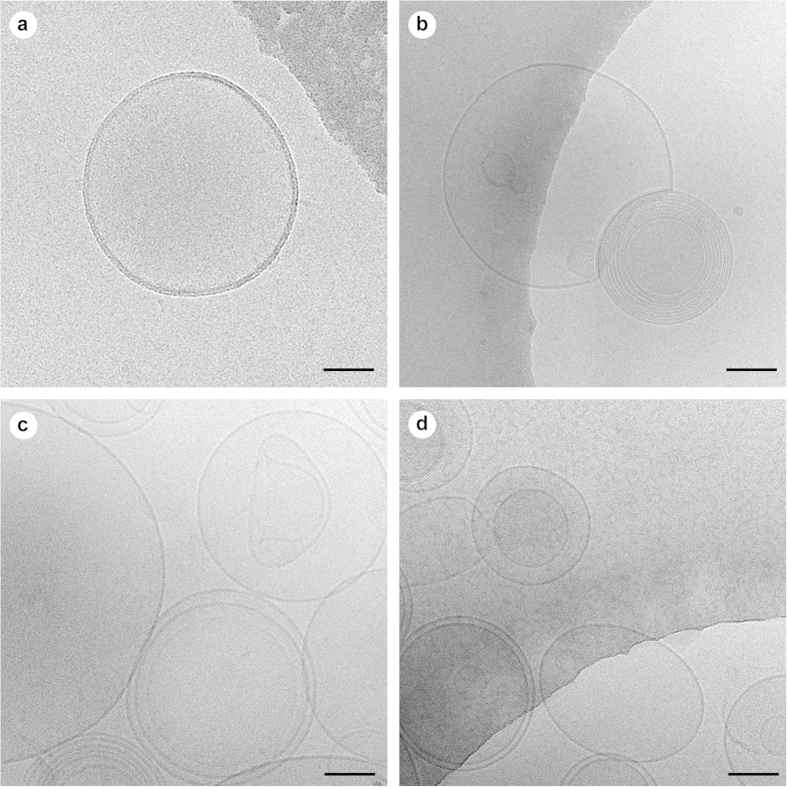
Cryo-EM ultrastructural characterization of EVs isolated from blood plasma by ultra-cushion and PROSPR methods. A heterogeneous population of EVs was observed in both methods including single layered vesicles, multilayered vesicles, exosomes (~100 nm) and microvesicles (>100 nm.) isolated by ultra-cushion (**a**,**b**) and PROSPR (**c**,**d**). Higher density of EVs was observed in PROSPR preparations including an odd-shaped vesicle as shown in micrograph (**c**). Scale bars in all images represent 55 nm.

**Figure 4:**
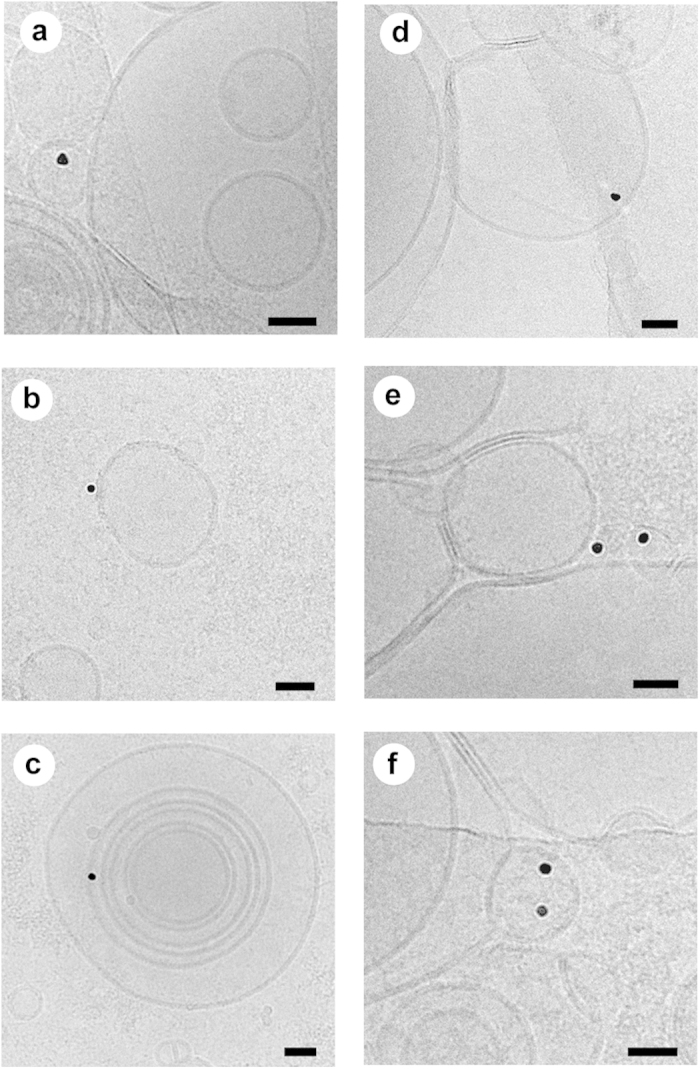
Cryo-immunogold EM micrographs of PROSPR isolated EVs. Presence of tetraspanin CD9 (10 nm colloidal gold particles) was observed as attached to the internal/external face of the membrane in single layered exosome-like vesicles (**a**,**b**) and in multilayered exosome-like vesicles (**c**). Presence of the ESCRT protein Alix was also confirmed (10 nm colloidal gold particles) in exosome-like vesicles isolated by PROSPR from blood plasma (**d**–**f**). Scale bars in all images represent 40 nm.

Having established that PROSPR is an efficient method for the separation of EVs from human blood plasma, we next proceeded to assess the structural integrity and diversity of the vesicles obtained using this method. Cryo-electron microscopy was used to determine the ultra-structural morphology of PROSPR-separated EVs for comparison with vesicles isolated by a standard ultra-cushion protocol. Using this approach, we observed that both PROSPR and ultra-cushion techniques generated a heterogeneous mix of circular, membrane-encapsulated structures with diameters ranging from 20–300 nm (Fig. 3). The morphologies and sizes of the structures we detected were consistent with those widely reported for exosomes (50–100 nm) and microvesicles (100–1000 nm)^14^. In addition, both the PROSPR and ultra-cushion methods yielded multi-layered and elongated vesicles of various sizes^15^, and separated non-membranous particles of ~30 nm diameter that resembled lipoproteins in size and morphology^15^,^16^,^17^,^18^. Although there was no difference in EV size distribution between the two methodologies tested here (data not shown), PROSPR separation appeared to generate a higher density of EVs. In order to obtain definitive evidence that PROSPR is an efficient method of EVs purification, we next conducted a confirmatory experiment using cryo-immunogold labeling to confirm the presence of characteristic EV markers (Alix and CD9) in PROSPR-separated EVs. Visualization of the immunogold-labeled EVs obtained by PROSPR confirmed and validated the presence of membrane-associated Alix and CD9 proteins on both multi-layered and single-layer EVs (Fig. 4). These data indicate that the plasma EVs obtained by PROSPR separation exhibit size distribution and biochemical features previously shown by those of EVs isolated using conventional techniques.

### Comparison of EV-derived proteins obtained by PROSPR versus sucrose ultra-cushion

**Figure 5:**
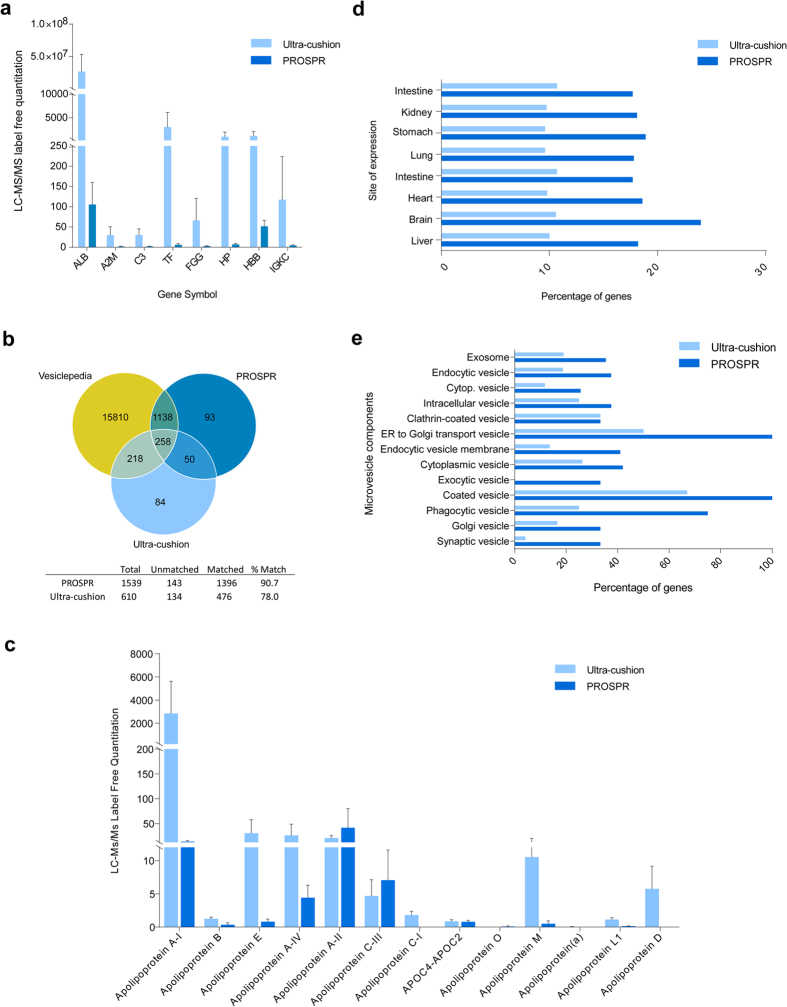
PROSPR generates higher purity plasma EVs as opposed to conventional ultra-cushion methods. LC-MS/MS label-free quantitation revealed that PROSPR separation yields high purity microvesicle fractions containing lower levels of contaminating plasma proteins when compared with samples obtained by ultra-cushion (**a**). The list of EV proteins identified by LC-MS/MS in ultra-cushion and PROSPR isolated fractions were compared with the list of human EV proteome contained in vesiclepedia^20^ as shown by the venn diagrams (**b**). Percentage of match between ultra-cushion and PROSPR was analyzed; PROSPR revealed higher percentage of match 90.7% compared to ultra-cushion 78.0% with vesiclepedia EV proteins. Cross-contamination in ultra-cushion and PROSPR isolated EV between proteins and high density lipoproteins was analyzed (**c**) the lipoproteins A-II and C-III were found at higher levels in PROSPR EV fractions whereas the lipoproteins A-I, E, A-IV, M and D were found at higher levels in ultra-cushion EV fractions. EV protein datasets were subjected to functional enrichment analyses using the FunRich tool^21^. Higher percentage of genes was identified in PROSPR fractions compared to ultra-cushion regarding site of expression (**d**) and analyzed microvesicle components (**e**).

High concentrations of unwanted plasma proteins in ultra-centrifuged samples are likely to impede the identification of EV-associated proteins and mRNAs with potential for use as disease biomarkers^19^. We therefore proceeded to use LC-MS/MS label-free proteomics to evaluate the relative levels of contaminating proteins present in the EV fractions obtained by ultra-cushion and PROSPR. When compared with the EV fraction obtained by ultra-cushion, we observed that the PROSPR-separated EV fraction exhibited dramatically reduced levels of serum albumin (ALB) and lower quantities of other plasma high abundant proteins (Fig. 5a). Indeed, albumin is the most abundant protein in human plasma, and the PROSPR-separated EV fractions contained less than 1% of the albumin levels present in the ultra-cushion purified fractions. When subsequently analyzed using the high-throughput Orbitrap mass spectrometers Elite and QExactive (Thermo Scientific Inc., Bremen, Germany), we detected a total of 1539 proteins in the PROSPR-isolated fraction, and a total of 610 proteins in the ultra-cushion EVs fraction (Supplemental Data Set 1). We then compared the percentage of match of these respective obtained datasets with Vesiclepedia, an extensive database containg proteins previously identified in isolated EVs^20^. The PROSPR-isolated fractions exhibited 90.7% of match with vesiclepedia and the ultra-cushion isolated fractions exhibited 78.0% of match (Fig. 5b). A total of 1396 EV proteins were identified in common between PROSPR and vesiclepedia and a total of 476 EV proteins were identified in common between ultra-cushion and vesiclepedia.

Isolation of EVs by ultra-cushion often results in co-isolation of high-density lipoproteins; accordingly we have analyzed the presence of these proteins in the PROSPR and ultra-cushion EV isolated fractions. Of the most abundant lipoproteins the A-II and C-III were highly present in PROSPR whereas ultra-cushion exhibited higher levels of apolipoproteins A-I, E, A-IV, M and D (Fig. 5c). Globally, these data indicated that PROSPR separation yields higher purity plasma EVs as opposed to conventional approaches. This was consistent with our earlier Western Blot results determining that PROSPR-separated fractions contained substantial levels of characteristic EV markers including CD9, CD63, Alix (Fig. 2c,d) and CD81 (Supplementary Figure 1).

We next sought out to elucidate if there were any significant differences in the EV-associated proteins/genes identified by LC-MS/MS when using PROSPR and ultra-cushion separated EVs. We performed a functional enrichment analysis of both datasets using FunRich^21^, an open source software which considers data from EV specialized databases including Vesiclepedia and Exocarta^20^,^22^. The percentage of gene identifications in PROSPR clearly outperformed those in ultra-cushion in all the analyzed categories (Fig. 5d,e). Of note, the higher percentage of exosome and brain proteins identified in PROSPR isolated EVs including several specific synaptic proteins. Furthermore, the percentage of proteins attributable to *coated vesicles* and *ER to golgi* categories which matched from PROSPR the 100% of the gene background lists in Funrich. Altogether it can be established that the EVs obtained by PROSPR separation are highly consistent with those currently being used in ‘omics’-based biomarker discovery studies.

## Discussion

In our current study, we report a novel method of isolating extracellular vesicles (EVs) from human plasma by PRotein Organic Solvent PRecipitation (PROSPR), which is simpler and quicker to use as opposed to conventional purification methods, yielding high-purity EVs suitable for detailed proteomic analyses. While EV preparations obtained using centrifugation-based techniques often include high concentrations of contaminating plasma proteins, our approach comprising the use of organic solvents to remove unwanted soluble proteins by precipitation represents an au courant and effective method of isolating EVs in human plasma samples for clinical use.

We observed that EV isolation by PROSPR removes the vast majority of contaminating plasma proteins and unwanted aggregates that would otherwise complicate analysis of EV cargo. After the removal of contaminating proteins by solvent-based precipitation, the lipid-encapsulated EVs are left behind in suspension and can easily be separated by vacuum centrifugation or MWCO filtration by centrifugation. Unlike ultracentrifugation, the PROSPR protocol involves only a few steps and takes a total time of less than two hours to complete. Furthermore, it only requires an acetone solvent which is extremely safe, inexpensive and widely available. The PROSPR method is a suitable method for biomarker measurements of individual patients in diverse clinical settings. Observed to generate an abundant heterogeneous population of EVs from small volumes of blood plasma, PROSPR is a viable and favourable alternative to conventional methods.

Our optimized PROSPR protocol utilizes a vacuum concentrator to concentrate the EVs prior to analysis by LC-MS/MS. Alternatively, a 300 kDa molecular weight cut-off (MWCO) filter could be used instead to separate the EVs out of suspension and remove additional small molecules, salts and metabolites prior to imaging studies. The extensive removal of soluble proteins is crucial to the success of the PROSPR method of EV purification. Protein molecules in aqueous solution are stabilized by exposing their hydrophilic regions in aqueous medium under constant dielectric strength^23^, which allows acetone to promote the attraction of opposing charged ions and induce protein aggregation^24^. In addition, Crowell *et al*. demonstrated that acetone-based protein precipitation could be enhanced by the presence of salts, which are abundant in human biological fluids^25^. The ion-pairing effects of these salts are thus likely to contribute to the efficacy of EV isolation by PROSPR. Contrary to our initial conjectures, our ultrastructural analyses revealed that EV diversity, structural integrity and intravesicular cargo remain stable for extended periods in cold 80% acetone. As such, this suggests that the PROSPR method is likely to be suitable for a wide range of biomarker discovery studies and clinical applications.

The reliable and economical method approach discussed in this study is highly advantageous in the aspects of cost, time and labour as opposed to conventional isolation methods. The use of PROSPR would therefore significantly accelerate the study of plasma-derived EVs and the translation of these data into novel clinical tests.

## Materials and Methods

### Biological samples

Blood plasma samples from a total of 20 healthy subjects were obtained at the Singapore General Hospital and National University Hospital of Singapore by standard protocol. Heparin was added to fresh whole blood samples and these were immediately centrifuged at 4200 × g for 10 min at 22 °C. Plasma was then frozen at −150 °C until analysis. Informed consent was obtained from all participants prior to the donation of blood samples used in our study. All experimental procedures were approved by the Institutional Review Board at Nanyang Technological University and were performed according to institutional guidelines.

### Reagents and Antibodies

All reagents were purchased from Sigma-Aldrich (MO., USA) unless otherwise indicated. Secondary antibodies HRP-conjugated rabbit anti-mouse (sc-358914), HRP-conjugated goat anti-rabbit (sc-2030) and primary antibodies rabbit polyclonal against CD81 (sc-9158), mouse monoclonal against CD9 (sc-13118) and mouse monoclonal against CD63 (sc-365604) were purchased from Santa Cruz Biotechnology (CA, USA). The mouse polyclonal antibody against Alix (ab88746) was purchased from Abcam (Cambridge, UK.). Protease inhibitor cocktail tables were obtained from Roche (Basel, Switzerland) and sequencing-grade modified trypsin was obtained from Promega (WI, USA).

### PRotein Organic Solvent PRecipitation (PROSPR)

Isolation of Evs by solvent-based precipitation was performed by directly mixing 500–1000 μl of neat plasma with four times the volume of cold acetone (−20 °C). The mixture was vortexed for a few seconds and then centrifuged at 3000 × g for one minute.

**Figure 6:**
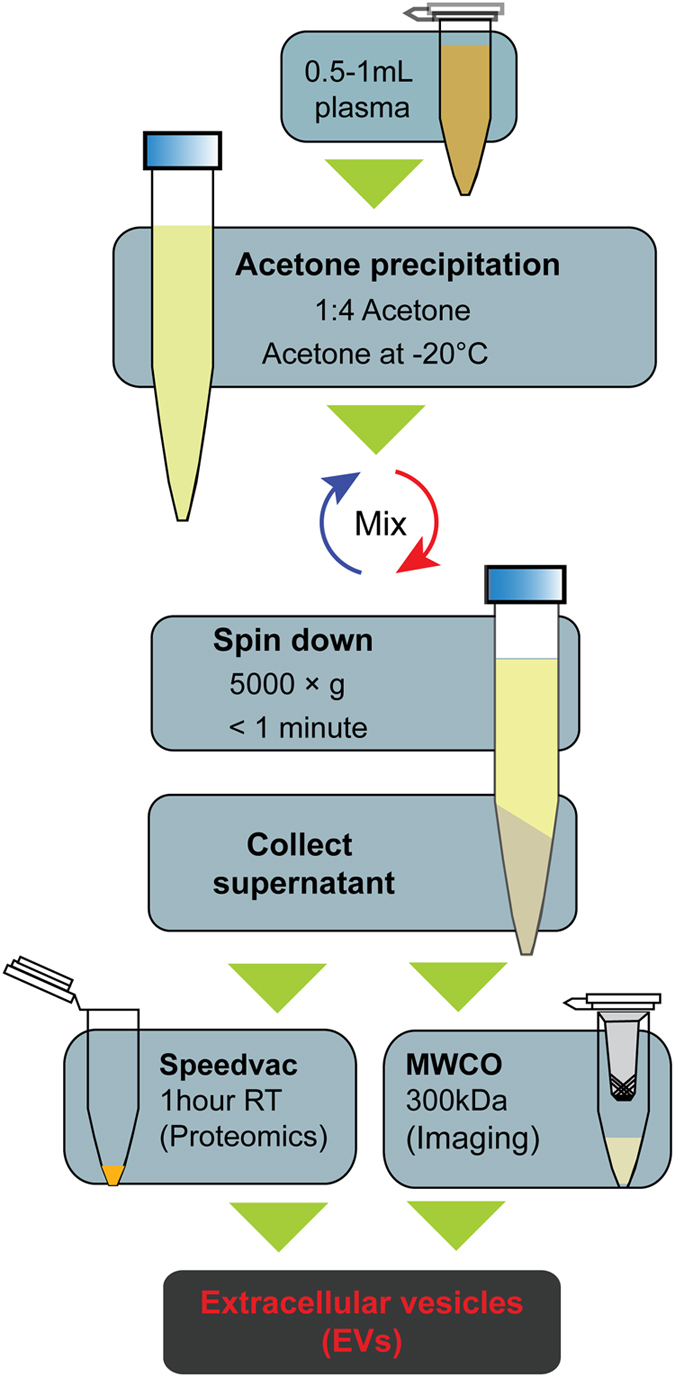
Schematic diagram depicting the method steps involved in the isolation of EVs from human blood plasma by PRotein Organic Solvent PRecipitation (PROSPR).

After centrifugation is completed, the pellet was either discarded or kept for further experiments. The EV-containing supernatant was removed and dehydrated in a vacuum concentrator (Concentrator Plus, Eppendorf AG, Hamburg, Germany) during approximately 1 hour. For imaging experiments the EV-containing supernatant was concentrated using a 300 kDa MWCO (see cryo-EM in the materials and methods section) for approximately 40 minutes (Fig. 6 **and** Supplementary Figure 1). The dried microvesicle fraction was resuspended and homogenized in bi-distilled water prior to be used in further analyses and the MWCO microvesicle fraction was directly used in further analyses.

### Microvesicle isolation by ultracentrifugation on a sucrose cushion (ultra-cushion)

Microvesicle isolation by ultracentrifugation onto a sucrose cushion was performed as previously described^26^,^27^,^28^,^29^,^30^,^31^,^32^ with some modifications for use with human plasma. Plasma was centrifuged at 300 × g, 4 °C for 30 minutes to remove cell debris. The resultant pellet was discarded, while the supernatant (S1) was centrifuged again at 16500 × g, 4 °C for 30 more minutes. The pellet from the second centrifugation step was removed and the supernatant (S2) was collected and mixed with chilled HPLC water (4 °C) to decrease sample density. The diluted supernatant was then transferred carefully into an ultracentrifuge tube containing 1 ml cold sucrose (5.5% solution chilled to 4 °C; approximate density 1.02 g/ml^33^) and centrifuged at 200,000 × g, 4 °C for 120 minutes to obtain the microvesicle pellet (P3) and supernatant (S3), which was then ultracentrifuged overnight (200,000 × g at 4 °C) to obtain the final pellet (P4).

### Western blot analysis

Microvesicles were transferred into lysis buffer (5% SDS in PBS) together with Complete Protease Inhibitor Cocktail (COMPLETE; Roche, Mannheim, Germany) and then boiled for 5 min at 95 °C under reducing conditions with 2-mercaptoethanol (5%) in BioRad 2× Laemmli sample buffer (CA, USA). Protein concentration was quantified by the Bradford assay and equal amounts of protein were used for Western blot analysis. To ensure that equal quantities of EVs were used in each experimental condition, EVs were first isolated from a pool of mixed plasma. The acetone fraction was later split equally for testing in each experimental condition. Proteins were subsequently resolved using either 10% or 15% SDS-PAGE and blotted onto nitrocellulose membranes. Protein detection was conducted using the appropriate primary antibodies at 1:1000 dilution (CD9, CD63, CD81 and Alix). Finally, appropiate HRP-conjugated secondary antibodies at 1:2000 dilution were used together with the Invitrogen ECL detection system (CA, USA).

### Ultrastructural studies of EVs

#### Cryo-electron microscopy

Microvesicles were PROSPR-separated from 1 ml total blood plasma. A 5 ml volume of the resultant EV-containing supernatant was later diluted in three-fold in 1× PBS, then concentrated 150-fold using a 300 kDa Pall Life Science Nanosep Centrifugal Device (MI, USA). EVs isolated by ultra-cushion were re-dissolved in 1× PBS and then concentrated similarly. The EV preparations were then directly deposited onto glow-discharged Quantifoil R2/2 grids coated with 2 nm carbon (Jena, Germany). Grids were blotted (1 second) at 95% humidity and plunge-frozen in liquid ethane using a Vitrobot plunger (FEI). Grids were imaged on an Arctica transmission electron microscope (FEI) operated at 200 kV on a Falcon II (FEI) direct electron detector. Images were recorded at −3 μm underfocus, with an electron dose of 15 e/Å^2^ and a nominal magnification of X 53,000. The object pixel size was 0.2 nm.

#### Cryo-immunogold EM labeling

Isolated microvesicles were deposited onto carbon-coated Quantifoil grids and washed with bi-distilled water in blocking solution (0.5% BSA/ddH_2_O) for 5 minutes. Samples were then incubated in a drop of primary antibody (CD9/Alix, 1:100 dilution) for 20 minutes. The grids were washed twice for 3 minutes in blocking solution, followed by adding 10 nm colloidal gold coated with a secondary antibody (goat anti-mouse 1:100 dilution, purchased from Aurion, Netherlands). Samples were washed twice again in bi-distilled water for 3 minutes and plunge-frozen as described above.

### Digestion of plasma EVs

Extracellular vesicles were lysed in 16 M urea, 50 mM ABB buffer together with Complete Protease Inhibitor Cocktail. Urea buffer was diluted to 8 M urea, 25 mM ABB with bi-distilled water prior to put the samples in reducing conditions. Proteins were then reduced in a final concentration of 10 mM dithiothreitol (DTT) for 3 h at 30 °C and alkylated using a final concentration of 20 mM IAA during 45 min at room temperature in the dark. After alkylation, urea concentration was diluted to <1 M using 25 mM ABB and digested overnight at 30 °C with trypsin at 1:50 dilution. Tryptic digestion was stopped by acidification diluting the digested samples to a 0.5% final concentration of FA. Peptides were reconstituted and desalted using Waters Sep-pack 50 mg C18 cartridges (MA., USA) performing several washes of 0.1% trifluoroacetic acid (TFA). Elution was carried out using 75% acetonitrile, 0.1% formic acid (FA), and the eluted samples were concentrated using vacuum concentrator.

### Fractionation of EV peptides

Desalted EV peptides were fractionated in order to decrease sample complexity. Briefly, samples were dissolved in 200 μL mobile phase A (85% ACN 0.1% acetic acid) and fractionated using a Fortis Amino column (4.6×200 mm, 3 μm, Fortis Technologies Ltd., Cheshire, UK) on a Shimadzu Prominence UFLC system monitored at 280 nm. Seventy-two fractions were collected with a 72 min gradient of mobile phase B (10% ACN, 0.1% FA), at 0% for 5 min, 0–20% B for 25 min, 20–33% B for 10 min, 33–60% B for 10 min, and 60–100% B for 5 min, followed by 17 min at 100% B at a flow rate of 1 ml/min. Fractions were dried to decrease the volume under vacuum and subsequently pooled according to peak intensities. Finally, peptides were completely dried and reconstituted with 3% ACN, 0.1% FA prior to be analyzed by mass spectrometry.

### LC-MS/MS spectrometry

LC-MS/MS analysis of digested peptides was performed on a Thermo Scientific Inc. Orbitrap Elite and QExactive mass spectrometers (Bremen, Germany) coupled with a Dionex UltiMate 3000 UHPLC system from Thermo Scientific Inc. Samples were sprayed using a Bruker-Michrom Inc. Michrom’s Thermo CaptiveSpray nanoelectrospray ion source (AL., USA). Approximately 2 μg of peptides were injected into a reverse phase Acclaim PepMap RSL column (Thermo Scientific; 75 μm ID × 15 cm, 2 μm particles) at a temperature of 35 °C and flow rate of 300 nl/min. Eluent A (0.1% FA in water) and eluent B (90% ACN/0.1% FA) were used to establish a 60 min gradient with elution starting at 3% eluent B for 1 min, which was then linearly increased to 35% eluent B over 47 minutes, before being increased to 50% eluent B over four minutes, and then increased to 80% eluent B in six seconds and kept isocratic for 78 seconds. Finally, the conditions were reverted to their initial state over six seconds and maintained for 6.5 minutes.

Thermo Scientific Orbitrap mass spectrometers were set to positive ion mode using LTQ Tune Plus software for data acquisition mode, alternating between a Full FT-MS (350–1600 m/z, resolution 60,000, with one μscan per spectrum) and a FT-MS/MS where the 10 most intense ions above a 500 count threshold were selected for fragmentation in high-energy collisional dissociation (HCD) mode (32% normalized collision energy, 150–2000 m/z, resolution 15,000, one μscan per spectrum, capillary temperature 250 °C, source voltage 1.5 kV). The automatic gain control target for FT-MS and MS/MS was set at 1e+06 and precursor ion charge state screening was activated.

### Bioinformatics and data analysis

Raw MS/MS data were de-isotoped and converted into Mascot Generic Format (mgf) files using Thermo Proteome Discoverer (version 1.4.1.14, Thermo Fisher Scientific Inc). Database searches were performed using an in-house Mascot server (version 2.3.02, Matrix Science, MA, USA) with a precursor MS tolerance of 10 ppm and MS/MS fragment tolerance of 0.02 Da. A false discovery rate (FDR) correction of 1% was applied to the identified peptides to enhance the confidence level of identified proteins (Supplementary Figure 2). The Mascot reported exponentially modified protein abundance index (emPAI) was used for protein quantitation as previously described^34^. Western blot signal was quantified by ImageJ software^35^. Student’s *t* test analyses were performed between PROSPR and ultra-cushion data using GraphPad Prism 6 (CA., USA) and *p*-values < 0.05 were considered significant unless specified otherwise. Error bars indicate SEM.

### Data Deposition

Proteomics data have been deposited to the ProteomeXchange Consortium^36^ via the PRIDE partner repository with the following identifier PXD002668.

## Additional Information

**How to cite this article**: Gallart-Palau, X. *et al*. Extracellular vesicles are rapidly purified from human plasma by PRotein Organic Solvent PRecipitation (PROSPR). *Sci. Rep*. **5**, 14664; doi: 10.1038/srep14664 (2015).

## Supplementary Material

Supplementary Figure S1

Supplementary Figure S2

Supplementary Dataset S1

Supplementary Dataset S2
