# Supplementary material for: Extracellular vesicles are rapidly purified from human plasma by PRotein Organic Solvent PRecipitation (PROSPR)
Source: Sci Rep. 2015 Sep 30;5:14664. doi: 10.1038/srep14664 (PMC4588595; doi:10.1038/srep14664)
Supplement: Supplementary Figure S1 [file srep14664-s1.pdf]

Extracellular vesicles are rapidly purified from human plasma by PRotein Organic Solvent PRecipitation (PROSPR)

Xavier Gallart-Palau, Aida Serra, Andrew See Weng Wong, Sara Sandin, Mitchell K.P. Lai, Christopher P. Chen, Oi Lian Kon, Siu Kwan Sze

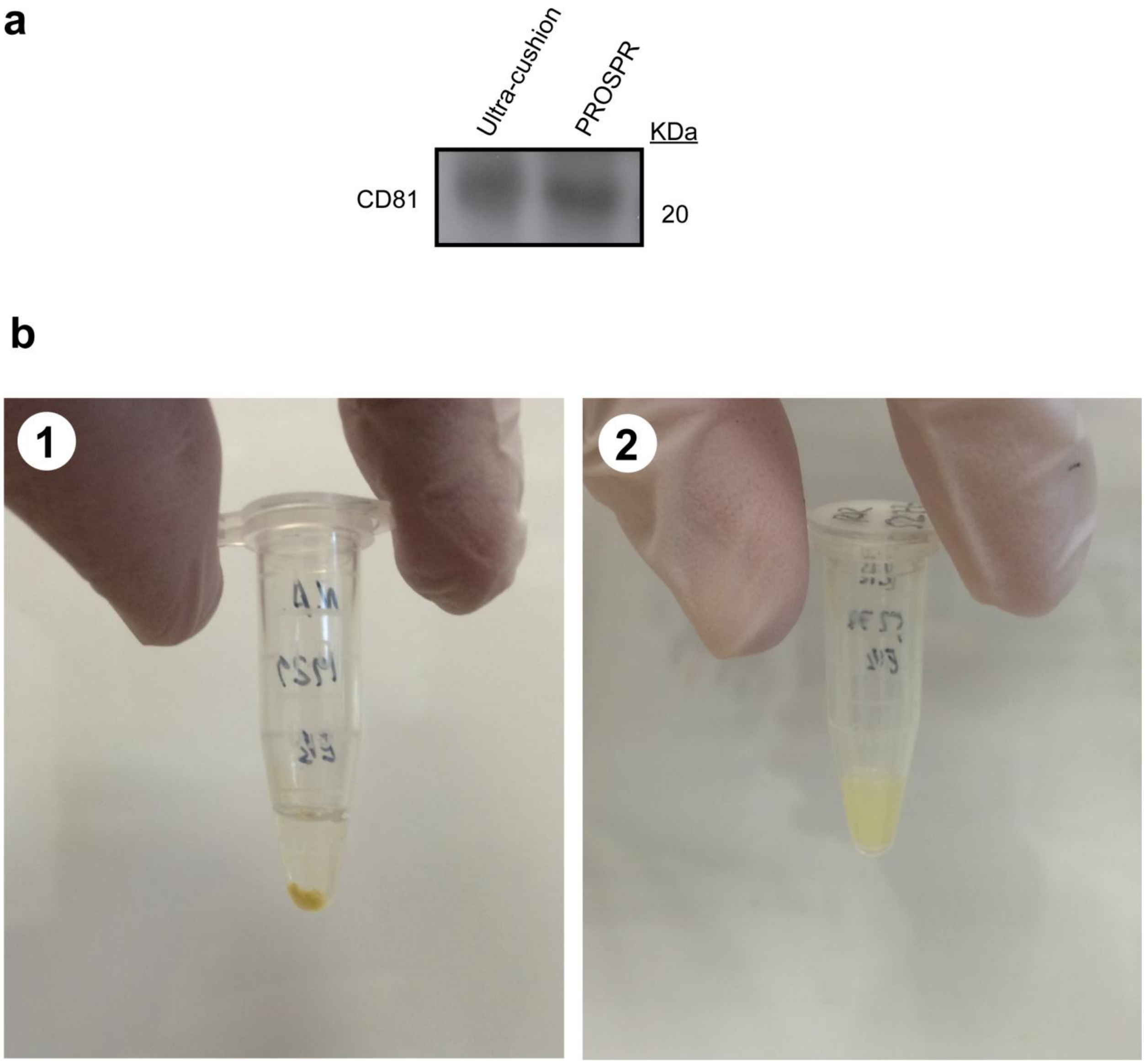

Supplementary Figure 1: (a) Western blot showing the presence of CD81 in plasma EVs isolated by ultra-cushion and PROSPR methods. (**b1**) The pellet containing separated EVs after vacuum evaporation of PROSPR supernatant is shown after addition of bi-distilled water. (**b2**) PROSPR EVs pellet resuspended in bi-distilled water.
