# Supplementary material for: Extracellular vesicles are rapidly purified from human plasma by PRotein Organic Solvent PRecipitation (PROSPR)
Source: Sci Rep. 2015 Sep 30;5:14664. doi: 10.1038/srep14664 (PMC4588595; doi:10.1038/srep14664)
Supplement: Supplementary Figure S2 [file srep14664-s2.pdf]

Extracellular vesicles are rapidly purified from human plasma by PRotein Organic Solvent PRecipitation (PROSPR)

Xavier Gallart-Palau, Aida Serra, Andrew See Weng Wong, Sara Sandin, Mitchell K.P. Lai, Christopher P. Chen, Oi Lian Kon, Siu Kwan Sze

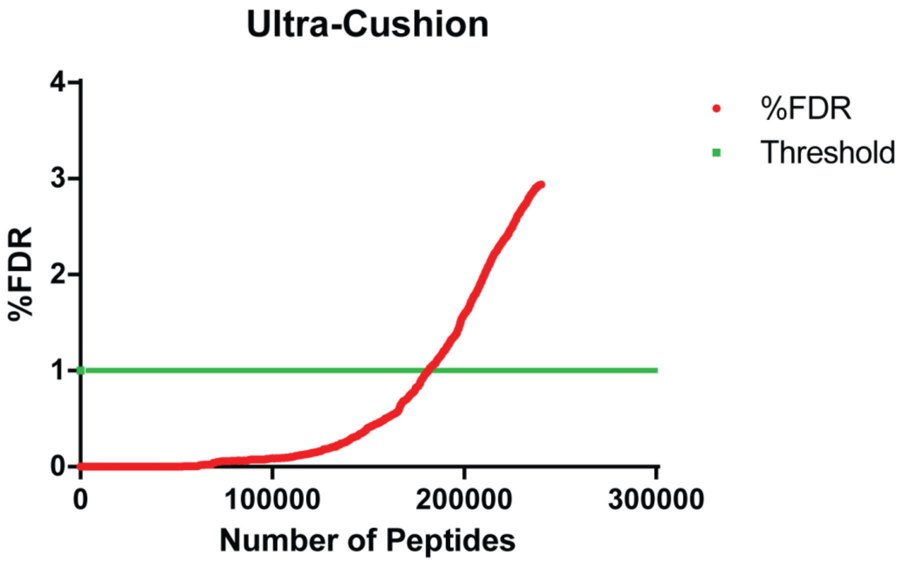

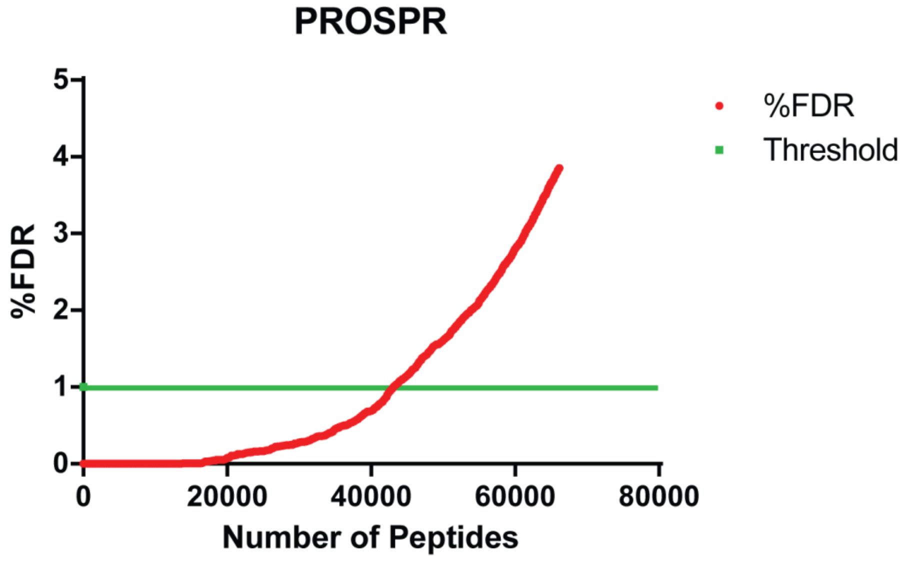

Supplementary Figure 2: FDR distribution of peptides identified by Ultra-cushion and PROSPR.
